# Comparison of Electroencephalography in Patients With Seizures Caused by Neurosyphilis and Viral Encephalitis

**DOI:** 10.3389/fneur.2022.879643

**Published:** 2022-05-27

**Authors:** Li-Li Zheng, Jing-Zhen Chen, Xiao-Rong Zhuang, Jia-Yin Miao

**Affiliations:** Department of Neurology, Zhongshan Hospital, Xiamen University, Xiamen, China

**Keywords:** neurosyphilis, encephalitis, viral, seizures, electroencephalography

## Abstract

**Background:**

Neurosyphilis (NS) lacks specificity in clinical and imaging features, and patients are frequently misdiagnosed as viral encephalitis when they present with seizures. This study aimed to compare electroencephalography (EEG) in patients with seizures resulting from the two diseases and provide guidance for differential diagnosis.

**Methods:**

A retrospective study on patients diagnosed with neurosyphilis and viral encephalitis with seizures in the Department of Neurology, Zhongshan Hospital, Xiamen University from 2012 to 2020.

**Results:**

A total of 39 patients with seizures caused by neurosyphilis and 40 patients with seizures caused by viral encephalitis were included. Chi-square test analysis showed that compared with patients with viral encephalitis, patients with neurosyphilis mainly developed in middle-aged and elderly people (*p* < 0.001), were more likely to have temporal epileptiform discharges (*p* < 0.001), and less likely to have status epilepticus (SE) (*p* = 0.029). There was difference between two groups in the EEG performance of lateralized periodic discharges (LPDs) (*p* = 0.085). The two groups were matched for age and sex by case-control matching, and 25 cases in each group were successfully matched. Patients with neurosyphilis were more likely to have temporal epileptiform discharges than those with viral encephalitis (*p* = 0.002), and there were no significant differences in LPDs (*p* = 0.077) and SE (*p* = 0.088) between two groups.

**Conclusion:**

When EEG shows temporal epileptiform discharges, especially in the form of LPDs, we should consider the possibility of neurosyphilis.

## Introduction

The high incidence of syphilis is a global health problem. It is estimated that 10 million people are infected each year worldwide, with a global epidemic rate of 0.5% (1, 2). Neurosyphilis can occur at any stage of syphilis infection and may lead to irreversible damage in the late stages of the disease. Therefore, early diagnosis and treatment of neurosyphilis are the keys to good prognosis. However, the clinical manifestations and imaging of neurosyphilis are variable and known as “great imitators,” especially in the presence of seizures, which can mimic the features of viral encephalitis and be misdiagnosed (3, 4).

The seizure frequency of neurosyphilis varies from 14 to 60%, and 10% of the patients experience reversible lateralized periodic discharges (LPDs) in the electroencephalogram (5, 6). The acute phase of viral encephalitis often presents with seizures, which occur in 40–65% of patients with herpes simplex encephalitis (HSE) (7). LPDs can appear on the electroencephalogram in more than 80% of HSE patients (8). The high frequency of seizures due to neurosyphilis and viral encephalitis is hard to differentiate by clinical presentation, imaging, or even cerebrospinal fluid examination. Therefore, this study aimed to compare electroencephalography (EEG) in patients with seizures resulting from the two diseases and provide guidance for differential diagnosis.

## Materials and Methods

### Study Design

This was a retrospective study on patients diagnosed with seizures caused by neurosyphilis and viral encephalitis who underwent video-EEG examination within the 1st week after seizures and received treatment in the Department of Neurology, Zhongshan Hospital Affiliated to Xiamen University between 2012 and 2020. The relevant data such as clinical and EEG characteristics of the patients were extracted.

### Study Subjects

Inclusion criteria: (1) met diagnostic criteria of neurosyphilis and viral encephalitis according to the 2014 European guidelines for the management of syphilis (9) and 2021 Chinese Guidelines for the Diagnosis and Treatment of Neurosyphilis (10). The diagnostic criteria for neurosyphilis were: positive serological test for Treponema pallidum; the presence of neurological symptoms or signs; cerebrospinal fluid (CSF) white blood cell count ≥5 × 10^6^/L, protein >500 mg/L; CSF toluidine red unheated serological test (TRUST) and/or positive serum treponema pallidum gelatin agglutination assay (TPPA). Viral encephalitis refers to the acute inflammatory process of the brain parenchyma caused by a direct viral infection, and patients with suspected encephalitis who meet any of the followings were diagnosed as viral encephalitis (11): positive virus-specific antibodies in serum or CSF; positive polymerase chain reaction (PCR) in CSF; positive viral culture in CSF; confirmed by histopathological examination; 4-fold increase in virus-specific antibodies in serum or CSF. According to this standard, we selected 40 patients with viral encephalitis confirmed by virus-specific antibodies and PCR in CSF, 15% of the patients were positive for both HSV-1 or HSV-2 antibodies and PCR; 55% were negative for antibodies but positive for PCR, and EEG LPDs were unremarkable; the rest are other types of pathogens such as EB virus and varicella-zoster virus, and the proportion of each type was low. (2) had epileptic seizures before or during admission: Epileptic seizures is a transient clinical manifestation caused by abnormal excessive and synchronized discharge activity of brain neurons. Epileptic seizures can be identified and classified based on their clinical and/or electroencephalographic features. (3) completed EEG and CSF examination within 1 week of seizures.

Exclusion criteria: (1) history and family history of epilepsy; (2) HIV infection; (3) other causes of secondary epilepsy.

### Clinical Parameters

Clinical parameters included basic demographic characteristics (age, gender), initial symptoms, seizure type, and the presence of status epilepticus (SE), which was defined according to the 2015 Report of the ILAE Task Force on Classification of Status Epilepticus (12). Seizure types were classified according to the International League Against Epilepsy (ILAE) Basic Edition 2017 Classification of Epilepsy (13). Since temporal epileptiform discharges are often accompanied by automatism and impaired awareness, our cases were finally divided into two categories: “focal progression to tonic-clonic seizures” and “focal motor seizures with impaired awareness.”

### Electroencephalographic Assessments

An internationally accepted 10–20 electrode placement system was used for all EEG studies. All patients had one or more video EEG recordings for at least 3 h. The EEG assessor used a blind method for the patient's clinical or imaging data. The recorded results included temporal epileptiform discharges, rhythmic focal slowing, LPDs, degree of abnormal EEG, posterior dominant rhythm, proportion of delta activities and spike and wave index (SWI). Proportion of delta activities refer to the proportion of delta activities in the background slowing and SWI refers to the percentage of spike and wave in the total non-rapid eye movement sleep time. Abnormal EEG was graded according to the Cleveland Epilepsy Center's EEG diagnostic grading standard as mild, moderate and severe. Mild abnormal EEG refers to non-specific slight slowing of the background. Moderate abnormal EEG refers to non-specific slow activity including focal intermittent slow activity and continuous diffuse slow activity mainly theta activities. Severe abnormal EEG refers to specific abnormality, such as epileptiform patterns, coma patterns, focal or diffuse continuous slow activities mainly delta activities. The above three indicators were all evaluated by two experienced electroencephalographers, who were blinded to clinical data, and in case of disagreement, the final results were reached by consensus. When focal slowing occurs with a similar frequency pattern, we call it rhythmic focal slowing.

### Statistical Analysis

Statistical analysis was performed using SPSS Statistics 26 software. Measurement data were expressed by mean (standard deviation) or median (interquartile range) according to whether they were normally distributed; categorical data were expressed by counts (proportion). Normally distributed and non-normally distributed variables were distinguished using the Test di Kolmogorov-Smirnov, and normally distributed variables were compared using the Student's *T*-test and non-normally distributed variables using the Mann-Whitney *U*-test. Differences in categorical variables were assessed using the chi-square test and the Mann-Whitney *U*-test. *P*-values < 0.05 were considered statistically significant. Age and gender were matched using case-control matching, and age-matched tolerance of 15 and a gender of 0 were set.

## Results

### Clinical Parameters

Of the 39 patients with seizures caused by neurosyphilis, 25 (64.1%) were male, with a mean age of 58.31 ± 12.5 years old, and of the 40 patients with seizures caused by viral encephalitis, 25 (62.5%) were male, with a mean age of 37.98 ± 20.2 years old. There was a significant difference in the age distribution between the two groups (*p* < 0.001), neurosyphilis patients were mainly middle-aged and elderly, and viral encephalitis patients had onset mainly in young and middle-aged adults; the proportion of SE in patients with viral encephalitis was 30% (12/40), significantly higher than 10.3% (4/39) in patients with neurosyphilis (*p* = 0.029; Table 1).

**Table 1:** Comparison of clinical characteristics of patients with seizures caused by neurosyphilis and viral encephalitis.

	**Neurosyphilis (*n* = 39)**	**Viral encephalitis ** **(*n* = 40)**	***P*-value**
**Demographic characteristics**			
Gender, male, *n* (%)	25 (64.1)	25 (62.5)	0.883
Age ($\bar{X}\;\pm$ SD), years	58.31 ± 12.5	37.98 ± 20.2	0.000^*^
**The first symptom**, ***n*** **(%)**			0.296
Epileptic seizure	25 (64.1)	21 (52.5)	
Other	14 (35.9)	19 (47.5)	
**Seizure type**, ***n*** **(%)**			0.115
Focal progression to tonic-clonic seizures	25 (64.1)	32 (80.0)	
Focal motor seizures with impaired awareness	14 (35.9)	8 (20.0)	
Status epilepticus, *n* (%)	4 (10.3)	12 (30.0)	0.029^*^

*$\bar{X}$, Arithmetic mean; SD, standard deviation*;

* *p < 0.05*.

### EEG Characteristics

**Figure 1:**
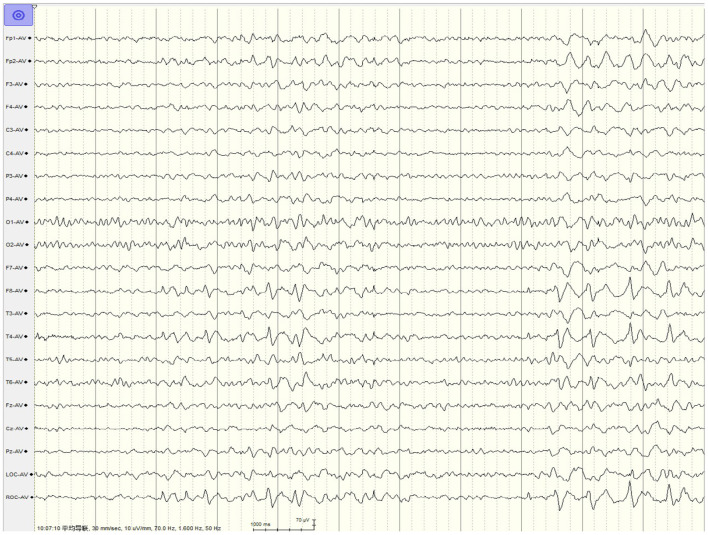
EEG in a 37-year-old male patient with neurosyphilis. LPDs were predominant in the right temporal region, appearing at a 1.5-Hz cycle.

The interictal EEG collected showed that the discharge sites of patients with seizures caused by neurosyphilis were mainly located in the temporal region, accounting for up to 89.7% (35/39), compared with 50.0% (20/40) of patients with viral encephalitis, and the difference was statistically significant (*p* < 0.001). The typical LPDs were shown in Figure 1 and the proportion of LPDs in the EEG of neurosyphilis patients was 46.2% (18/39), which was higher than that of viral encephalitis patients of 27.5% (11/40) but did not reach statistical significance (*p* = 0.085; Table 2).

**Table 2:** Comparison of EEG characteristics between patients with seizures caused by neurosyphilis and viral encephalitis.

	**Neurosyphilis** ** (*n* = 39)**	**Viral encephalitis ** **(*n* = 40)**	***P*-value**
Temporal epileptiform discharges, *n* (%)	35 (89.7)	20 (50.0)	0.000^*^
LPDs, *n* (%)	18 (46.2)	11 (27.5)	0.085
Rhythmic focal slowing, *n* (%)	19 (48.7)	24 (60.0)	0.314
**Posterior dominant rhythm**, ***n*** **(%)**			0.645
Decrease	23 (59.0)	25 (62.5)	
Absent	12 (30.8)	13 (32.5)	
Normal	4 (10.3)	2 (5.0)	
**Degree of abnormal EEG**, ***n*** **(%)**			0.673
Mild	14 (35.9)	12 (30.0)	
Moderate	13 (33.3)	15 (37.5)	
Severe	12 (30.8)	13 (32.5)	
Proportion of delta activities (%) (M, IQR)	30, 10–50	22.5, 5–50	0.587
Spike and wave index (%) (M, IQR)	30, 5–40	15, 5–30	0.249

*LPDs, lateralized periodic discharges; M, median; IQR, interquartile range*.

* *p < 0.05*.

### Matching Analysis

Two groups of patients after matching for age and gender were further analyzed, and 25 cases in each group were successfully matched. The results showed that patients with neurosyphilis were more likely to have temporal epileptiform discharges than those with viral encephalitis (*p* = 0.002), and the *p*-value indicated no significant differences between the two groups in LPDs (*p* = 0.077) and SE (*p* = 0.088; Table 3).

**Table 3:** Comparison of patients with seizures due to neurosyphilis vs. viral encephalitis after age and sex matching.

	**Neurosyphilis (*n* = 25)**	**Viral encephalitis** ** (*n* = 25)**	***P*-value**
**Demographic characteristics**			
Gender, male, *n* (%)	18 (72.0)	18 (72.0)	1.000
Age ($\bar{X}\;\pm$SD), years	56.96 ± 12.3	48.64 ± 18.2	0.064
**The first symptom**, ***n*** **(%)**			0.087
Epileptic seizure	17 (68.0)	11 (44.0)	
Other	8 (32.0)	14 (56.0)	
**Seizure type**, ***n*** **(%)**			0.136
Focal progression to tonic-clonic seizures	14 (56.0)	19 (76.0)	
Focal motor seizures with impaired awareness	11 (44.0)	6 (24.0)	
Status epilepticus, *n* (%)	3 (12.0)	8 (32.0)	0.088
Temporal epileptiform discharges, *n* (%)	22 (88.0)	12 (48.0)	0.002^*^
LPDs, *n* (%)	12 (48.0)	6 (24.0)	0.077
Rhythmic focal slowing, *n* (%)	19 (48.7)	24 (60.0)	0.314
**Posterior dominant rhythm**, ***n*** **(%)**			0.825
Decrease	16 (59.0)	17 (62.5)	
Absent	6 (30.8)	6 (32.5)	
Normal	3 (10.3)	2 (5.0)	
**Degree of abnormal EEG**, ***n*** **(%)**			0.844
Mild	10 (40.0)	9 (36.0)	
Moderate	9 (36.0)	10 (40.0)	
Severe	6 (24.0)	6 (24.0)	
Proportion of delta activities (%) (M, IQR)	30, 10–47.5	20, 5–45	0.378
Spike and wave index (%) (M, IQR)	20, 5–35	10, 5–27.5	0.252

* *p < 0.05*.

## Discussion

In this retrospective analysis, we compared two groups of patients with seizures caused by neurosyphilis vs. viral encephalitis. EEG suggested that the two groups were significantly different in temporal epileptiform discharges. LPDs may be different, but the included sample size was not large enough to achieve power. These results suggest that when EEG shows temporal epileptiform discharges, especially in the form of LPDs, we should consider the possibility of neurosyphilis.

The location of EEG discharges and the origin of seizures can indicate the location of brain tissue damage and epileptogenic foci, while neurosyphilis can present with changes in medial temporal lobe signals in patients with seizures, which suggested the presence of temporal epileptiform discharges on the EEG (5, 14, 15). In this study, the proportion of patients with seizures caused by neurosyphilis with EEG discharge sites located in the temporal region reached 89.7% (35/39), suggesting that temporal lobe lesions should not be ignored in patients with seizures caused by neurosyphilis and may be much more common than currently reported. Patients with viral encephalitis, especially herpes simplex encephalitis, often present with the changes in the signal intensity of the medial temporal lobe parenchyma. Therefore, the significant difference in temporal area epileptiform discharges on the EEG may help differentiate the two diseases.

LPDs are consisted of repetitive, rhythmic lateralization or local spike and wave, and discharges are periodic, usually occurring periodically at a frequency of 1–2 Hz (16). LPDs mostly appear in patients with acute brain injury, especially in patients with acute stroke, tumors, or central nervous system infections (17). However, the proportion of patients with viral encephalitis experiencing LPDs in this study was low (11/40, 27.5%), probably because the number of patients positive for both HSV-1 or HSV-2 antibodies and PCR with herpes simplex encephalitis was relatively small (6/40, 15%). Further studies are needed to confirm our conclusion. Patients with LPDs have a seizure risk of 50–90% and are highly associated with most recent seizure, and most EEGs with LPDs have been reported to be obtained within 4 days of seizure activity or SE (18, 19). EEG examination is well-established within 1 week of seizure onset, which may explain why a higher proportion of patients with seizures caused by neurosyphilis experienced LPDs in this study (18/39, 46.2%). However, there is still a lack of large-scale clinical studies of LPDs in patients with neurosyphilis. In contrast, LPDs have been confirmed in viral encephalitis, while in this study patients with seizures caused by neurosyphilis were more likely to have LPDs than patients with viral encephalitis.

Neurosyphilis is divided into five types according to the site of involvement: asymptomatic NS, meninges NS, meninges/spinal membrane vascular syphilis, spinal tuberculosis, and paralytic dementia. The combined involvement of vasogenic, cell-derived edema caused by meningovascular inflammation and gliosis due to the infection-induced small vessel ischemic changes is one of the mechanisms of epilepsy in patients with neurosyphilis (20, 21). Therefore, meningovascular type is more common in patients with seizures (5). Meningovascular syphilis mainly affects large and medium-sized arteries, and eventual vascular occlusion leads to ischemia and infarction, while ischemic stroke is the most common cause of LPDs in adults. Therefore, LPDs can appear secondary to ischemia in meningovascular syphilis (22). Neurosyphilis is a chronic central nervous system disease. Treponema pallidum infection leads to chronic inflammatory environment in the brain. Therefore, under the combined action of acute and chronic brain damage, the proportion of LPDs in patients with neurosyphilis may be related to that in patients with viral encephalitis mainly with acute brain damage.

In this study, patients with seizures caused by viral encephalitis experienced significantly higher SE than patients with neurosyphilis. After matching for age and gender, the difference did not reach statistical significance (*p* = 0.088), suggesting that the difference in SE may be due to the confounding effect of age and may not necessarily be related to the type of disease. SE is rare in patients with neurosyphilis (5, 23), four patients (10.3%) with neurosyphilis experienced SE in this study. Snotgras et al. speculated that LPDs were part of the presentation of SE (24). The relationship between SE and the high proportion of LPDs in this group of neurosyphilis patients cannot be ruled out.

At present, there is no gold standard for the diagnosis of neurosyphilis, and the diagnosis depends on the comprehensive analysis of clinical manifestations, serological tests and cerebrospinal fluid tests (25). Therefore, it is urgent to find additional supportive criteria with differential significance for the diagnosis of neurosyphilis and viral encephalitis. The differential findings of EEG between the two diseases in this study can provide a reference value for clinical diagnosis and treatment.

This study has limitations such as retrospective study design, single center, and small sample size. In addition, the differential findings on electroencephalography did not yet provide differential significance between neurosyphilis and viral encephalitis. Failure to analyze other characteristics of the seizures between the two groups is another limitation of this study. LPDs are independent predictors of poor prognosis (26, 27). Further controlled, prospective, multicenter trials will better understand the link of LPDs with neurosyphilis, and provide reference value for exploring the differentiation of EEG between neurosyphilis and viral encephalitis.

In conclusion, EEG differs in patients with seizures due to neurosyphilis and viral encephalitis, especially on temporal area discharges. Although LPDs were not significantly different between the two diseases, it is suggested that when EEG shows temporal epileptiform discharges, especially in the form of LPDs, we should consider the possibility of neurosyphilis.

## Data Availability Statement

The original contributions presented in the study are included in the article/supplementary material, further inquiries can be directed to the corresponding author.

## Ethics Statement

The studies involving human participants were reviewed and approved by Xiamen University. The patients/participants provided their written informed consent to participate in this study.

## Author Contributions

L-LZ wrote manuscript. J-ZC and X-RZ completed data collection. J-YM designed and supervised the study. All authors reviewed the manuscript.

## Funding

This study was supported by the grants from National Natural Science Foundation (No. 81400984), Natural Science Foundation of Fujian Province (No. 2014D009), Natural Science Foundation of Fujian Province (No. 2020J011209), and Xiamen Medical and Health Guidance Project (No. 3502Z20214ZDI036).

## Conflict of Interest

The authors declare that the research was conducted in the absence of any commercial or financial relationships that could be construed as a potential conflict of interest.

## Publisher's Note

All claims expressed in this article are solely those of the authors and do not necessarily represent those of their affiliated organizations, or those of the publisher, the editors and the reviewers. Any product that may be evaluated in this article, or claim that may be made by its manufacturer, is not guaranteed or endorsed by the publisher.
